# Polymerization Inhibition by Triplet State Absorption for Nanoscale Lithography

**DOI:** 10.1002/adma.201204141

**Published:** 2013-01-09

**Authors:** Benjamin Harke, William Dallari, Giulia Grancini, Daniele Fazzi, Fernando Brandi, Annamaria Petrozza, Alberto Diaspro

**Affiliations:** Department of Nanophysics, Istituto Italiano di Tecnologia (IIT)Via Morego 30, 16163 Genova, Italy; Center for Nano Science and Technology@PoliMi, Istituto Italiano di Tecnologia (IIT)Via Giovanni Pascoli 70/3, 20133 Milano, Italy; Dipatimento di Fisica, Universita' degli Studi di Genovavia Dodecaneso 13, 16153, Genova, Italy

**Keywords:** stimulated emission depletion (STED), reversible saturable optical fluorescence transition (RESOLFT), isopropyl thioxanthone (ITX), 7-diethylamino-3-thenoylcoumarin (DETC), photoinduced absorption

In the field of photopolymerization, multi-photon lithography^1^ stands out as a mask-free tool for fabricating three-dimensional structures inside a photocurable resin that is transparent at the wavelength used. This approach enables a great deal of versatility compared with single photon absorption lithography where the thickness of the polymerized parts is limited by the penetration depth of the light in the resin^2^ and therefore a layer-by-layer approach is necessary to fabricate 3D structures.^3^ The possibility of producing 3D, free-standing, and arbitrarily complex architectures opens up novel applications in various research fields, e.g., photonic crystals, metamaterials, and biological scaffold fabrication.^4–10^

In two-photon lithography, also known as direct-laser-writing (DLW) lithography,^1^, ^6^ the spatial resolution is unfortunately restricted by diffraction that limits the performances of the fabrication system.

It has been shown^11–13^ that in a system with a very sharply defined polymerization threshold the extension of single isolated features, e.g., a line or a voxel, can be reduced without theoretical limit.^14^ A photocurable resin exposed to a light dose below this threshold will not cure and, hence, under near-threshold irradiation conditions, voxels with an extension well below the diffraction limit can be fabricated. Unfortunately, light doses below this threshold are also partially absorbed and create thereby a kind of *memory effect*^14^ in the resin. With any following exposure of the resin even below the threshold, an accumulation of the light doses will effectively lift the system above the threshold and also cause curing in underexposed areas. This effect imposes a limit on the minimum distance between two adjacent features, usually defined as the resolution of the system.

In other words, the presence of a threshold enables the fabrication of single isolated features with a sub-diffraction size while the resolution is constrained by the diffraction limit which can only be shifted by the use of non-linear effects, such as two-photon excitation and non-linear curing rates of the resin.

The addition of diffusing radical quenchers^15^, ^16^ in the resin can reduce the above-mentioned memory effect and lead to an improved resolution. Nevertheless, this approach requires slow scanning speeds which increase the fabrication time.

Another approach to significantly improve the resolution in DLW lithography is inspired by STED (stimulated emission depletion^17^) microscopy and has been investigated during the last years.^14^, ^18–20^ STED microscopy is the most prominent example of a more general concept for achieving sub-diffraction resolution that is called RESOLFT (reversible saturable optical fluorescence transition^21^). The basic conceptual idea of RESOLFT is to selectively switch between two photophysical states and thus confine light emission, e.g., fluorescence, or a chemical reaction, e.g., polymerization, to an active volume of sub-diffraction size. To this extent, the diffraction-limited sized excitation beam is overlaid with a second beam, the switching beam, which by means of interference effects^22^ is shaped so the light intensity distribution is zero in the center of the focal spot and maximum at its periphery. In this way, only molecules located in the surroundings of the excitation focus where the switching beam has a pronounced intensity value are switched back, while molecules located close to the centre are unaffected and remain in the excited state. Because of the saturation effect of the switching process, a higher switching intensity confines the area or volume of excited molecules even closer to the focal spot centre leading to sub-diffraction resolution.^23^ It is worth noting that the final resolution of the system is not theoretically limited by diffraction anymore, but it is strongly related to the switching efficiency.^24^

The possibility of shaping the focal volume in any spatial dimension^25^ without a theoretical limit enables fabrication of arbitrary structures within the volume of a photocurable resin. This was one of the reasons why the RESOLFT concept has become particularly interesting for the field of DLW lithography. In order to apply the RESOLFT concept to DLW, photoinitiator (PI) molecules must undergo a switching event between a chemically active state responsible for the polymerization and a chemically inert state.^26^ In the case of conventional PIs used in DLW, the photophysical state leading to polymerization has been attributed to a triplet state.^27^ Consequently, polymerization inhibition can either be achieved by avoiding the population of the triplet state or by effectively depopulating it. Since the resolution enhancement provided by RESOLFT mainly depends on the efficiency of the switching process, a very detailed understanding of the exact nature of the switching transition is required. Once the intramolecular transitions are determined, an optimization of the process can be carried out. This means that the wavelength of the switching laser can be tuned to the correct transition energy maximizing the switching cross section. Moreover, the knowledge of the lifetime of the involved state helps with the choice of the switching laser between pulsed and continuous wave (CW). In the case of long switching state lifetimes (microseconds), for instance, relatively inexpensive CW lasers can be used enabling maximum system performance at minimum cost. For these reasons, several studies have been performed^28–30^ to determine the process behind the polymerization inhibition. Although important information on the polymerization inhibition timing and the photophysical properties of the PIs have been reported, a clear picture of the mechanism driving the inhibition process is not available yet. In this work, we combine DLW high-end fabrication with nanosecond transient absorption spectroscopy and density functional theory (DFT) calculations to test a series of different PIs. This exceptionally broad set of investigation techniques allows us to demonstrate that the capability of inhibiting the polymerization process can be related, in a general way, to the depletion of the PIs’ lowest triplet state (T_1_) population when the switching beam is resonant to the (T_1_→T_*n*_) triplet state absorption (TSA) energies.

**Figure 1:**
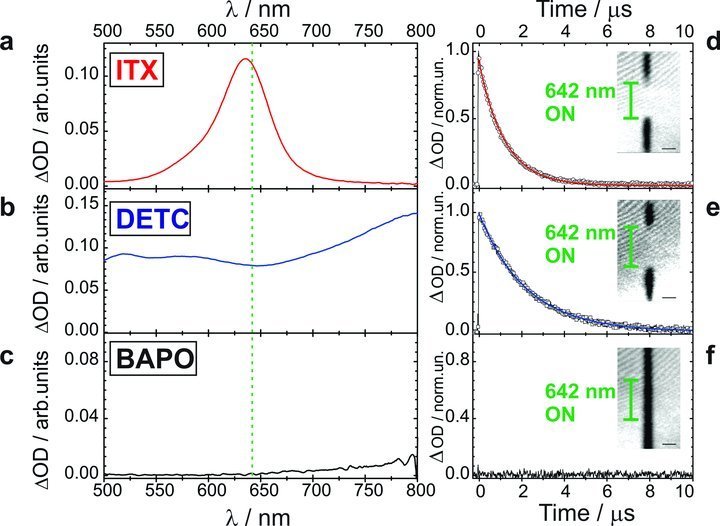
Investigation of three different PIs (ITX, DETC, and BAPO) by means of nanosecond transient absorption spectroscopy. a–c) PA spectra. d–f) Time dynamics at 642 nm. Insets of (d–f): confocal reflection measurements of a standard photopolymerization inhibition test (scale bar: 500 nm). All PIs were dissolved in the acrylic monomer PETA. In order to match the absorption spectrum of the different PIs, excitation was tuned between 415 and 425 nm (fluence ∼2 mJ cm^−2^).

To start our investigation, we performed transient absorption spectroscopy on several PIs gathering spectral and temporal information on the excited states in a nanosecond to millisecond time window. We then correlated these results with polymerization inhibition capability. Remarkably, the spectroscopy measurements were performed dispersing the PIs in the same resin (acrylic monomer pentaerythritol triacrylate, PETA) used during fabrication, thus ensuring an identical chemical environment. Isopropyl thioxanthone (ITX) and 7-diethylamino-3-thenoylcoumarin (DETC) are the PIs chosen to start our investigation. This is because polymerization initiated by these PIs has been shown to be inhibited by irradiation with a second light beam of a wavelength between 525 and 642 nm.^18^, ^29^, ^30^ We also tested phenylbis(2,4,6-trimethylbenzoyl)phosphine oxide (BAPO), a PI in which polymerization cannot be inhibited either by irradiation with light at 532 or at 642 nm.^18^, ^30^ **Figure** **1** shows the photoinduced absorption (PA) spectrum integrated over a time window of 500 ns and the PA dynamics probed at 642 nm for each of these PIs. Confocal reflection measurements in the insets show the capability of inhibiting the polymerization by simply polymerizing a single line with 1PE while the shutter of the inhibition laser is sequentially opened and closed (both foci Gaussian shaped). For ITX and DETC, the PA spectrum shows a positive fractional change in absorption (ΔOD/OD) that can be assigned to the absorption of a relatively long-living photoexcited species. In particular, ITX displays a well-defined absorption peak around 630 nm whereas DETC has a broad absorption from the visible to near-IR spectrum. The temporal dynamics show a decay time of the photoexcited species of ∼1.2 μs for ITX and ∼2.3 μs for DETC. The polymerized line in the right panels indeed shows the inhibition of polymerization initiated by these PIs by a 642 nm light beam. In the case of BAPO, neither the PA spectrum nor the time dynamic measurement showed a significant absorption within the response time of the instrument (∼4 ns). However, it should be noted that the existence of possible short lived species, which would prevent in any case an efficient polymerization inhibition by CW lasers, cannot be excluded.^31^ The same experiments have been performed using two alternative PIs, anthraquinone and camphorquinone (Sigma Aldrich) and no PA spectra have been seen (data not shown). Accordingly, no polymerization inhibition by illumination with CW 642 nm light has been observed for these three PIs.

The results shown in Figure 1 indicate a good correlation between the photon energy needed to inhibit the polymerization process and the transition energy of the photoinduced species. Moreover, it is worth noting that the lifetimes of the excited species found for ITX and DETC are in good agreement with the time scales reported for the polymerization inhibition dynamics^30^ of the same molecules.

**Figure 2:**
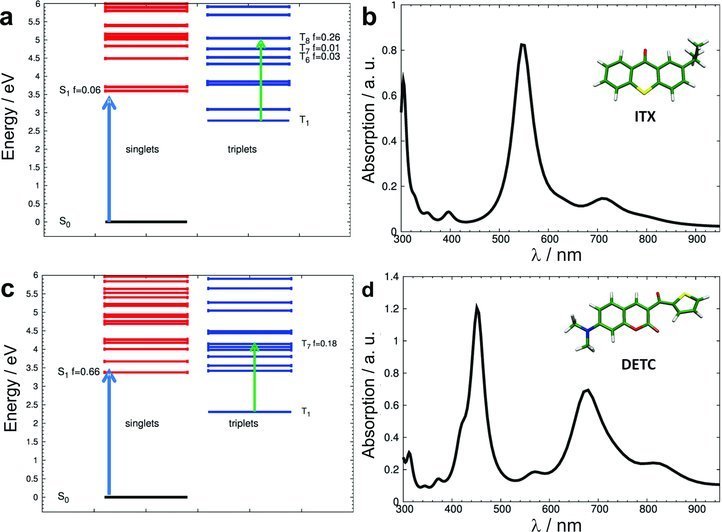
TD-(U)DFT vertical excited state transitions. Red lines S_0_→S_*n*_ electronic transitions, blue lines T_1_→T_*n*_ transitions for a) ITX and c) DETC. TD-UDFT triplet–triplet absorption spectra plotted by using a Lorentzian fit with a width of 200 cm^−1^ for a) ITX and c) DETC. The insets show DFT (PBE0/6-311G**) optimized molecular structures for ITX and DETC in their ground singlet state (S_0_) and triplet state (T_1_). Red atoms are oxygen, green carbon, white hydrogen, blue nitrogen, and yellow sulfur.

According to the literature,^32^ the absorption band observed in the visible range for ITX could be assigned to the PA within the triplet manifold (T_1_→T_*n*_). In order to clearly determine the nature of such a PA band, we performed quantum chemical DFT and time-dependent DFT (TDDFT) calculations of both ground and excited states with singlet and triplet spin multiplicity. In the insets of **Figure** **2**, the DFT (PBE0/6-311G**) optimized molecular structures for ITX and DETC in their ground state (S_0_) are shown. The corresponding TD(U)-PBE0/6-311G** singlet and triplet excited state energies are calculated on the relative equilibrium geometries for ITX (Figure 2a) and DETC (Figure 2c); the highest oscillator strength is highlighted in each of the energy diagrams for both singlet and triplet excited state transitions. While ITX presents a dominant (T_1_→T_8_) transition, DETC has a distribution of transitions with no negligible oscillator strength resulting in a broader triplet absorption spectrum. The relative simulated (T_1_→T_*n*_) absorption spectra are shown in Figure 2b and d.

Taking into account the level of DFT theory used and possible effects due to the chemical environment^33^ we can confidently say that the computed (T_1_→T_*n*_) absorption spectra are in good agreement with the measured PA spectra for both molecules. The combination of experimental and theoretical results indeed demonstrates that the long-lived photogenerated species are molecular triplet excitations, indicating that the switching beam can efficiently inhibit the polymerization process when it is resonant to (T_1_→T_*n*_) transitions with no negligible oscillator strength and the T_1_ state has a sufficiently long lifetime. The polymerization inhibition then occurs by TSA, mediated by the depletion of the lowest triplet state population acting as an intermediate species of the polymerization process. It is important to note here that, although this has been identified as the crucial mechanism which allows for polymerization inhibition, the corresponding depopulation channel still needs to be clarified. Since internal conversion in the triplet manifold (T_*n*_→T_1_) is a very fast process (∼hundreds of femtoseconds), a very efficient transition from the triplet manifold to a different molecular excitation, through a high energy triplet state, is required to observe polymerization inhibition in steady-state conditions. A possible mechanism, for example, could be based on an effective T_*n*_–S_*n*_ transition, i.e., reverse inter-system crossing.^34^

In order to demonstrate that TSA lithography can produce structures with an increased resolution relative to conventional DLW, we fabricated a periodic point pattern with a centre-to-centre distance of 250 nm. It should be noted that such periodicity is challenging for conventional DLW. Uniform resolution enhancement along the lateral plane was achieved by placing into the switching beam path a phase plate forming a donut-shaped intensity distribution in the focal plane.

**Figure 3:**
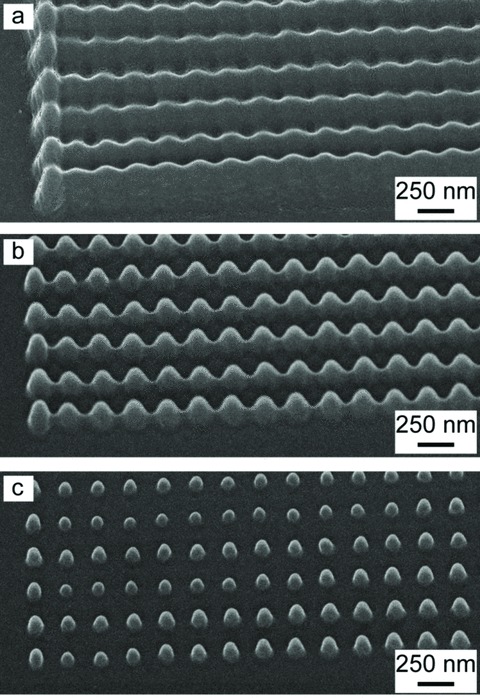
SEM images (tilted view) of three point patterns (periodicity: 250 nm, pixel dwell time: 0.3 ms) fabricated in a sample of DETC/PETA using different switching powers. The point grid is fabricated by conventional DLW lithography (a) and with TSA lithography at switching powers of 14 (b) and 48 mW (c). The excitation power was kept constant for each pattern (7 mW). All power values were measured at the back aperture of the objective lens.

The results are presented in **Figure** **3** as tilted scanning electron microscopy (SEM) images for conventional DLW lithography, and after applying 14 or 48 mW of inhibition light (full data set of the point pattern can be found in the Supporting Information). A high contrast and resolution are clearly visible even when low power switching light is applied: fabricated points become separated more clearly than in the case of conventional DLW. When the highest inhibition power is applied, the best results are achieved and a good separation of the fabricated points can be observed. These findings highlight the outstanding benefit that the fabrication system gains when applying the TSA lithography concept: without changing any parameter of the fabrication process, its performance is significantly enhanced, as shown by the fact that not only the size of a single isolated voxel is reduced but also close distance features (250 nm) are clearly separated.

**Figure 4:**
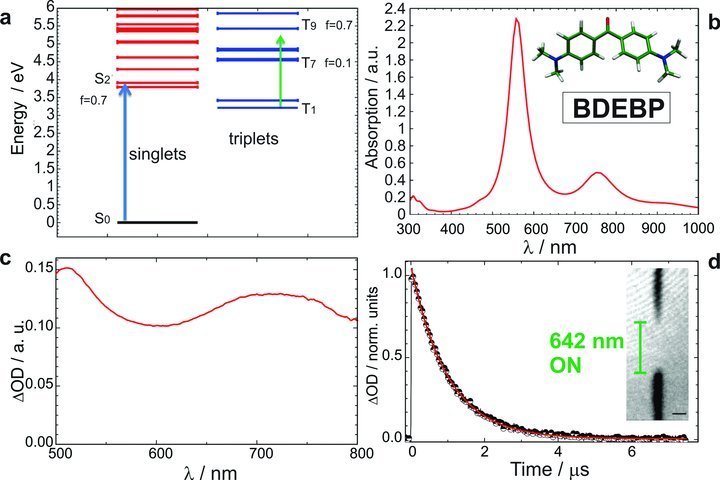
Theoretical and experimental investigation of an alternative PI, BDEBP, by means of nanosecond TAS and DFT. Top row from left to right: PA spectrum, time dynamics with 642 nm probe light and polymerization inhibition test (scale bar: 500 nm). Bottom row from left to right: chemical structure at ground energy state, energy level with corresponding oscillator strengths and theoretical triplet spectrum.

Finally, knowing the photophysical reaction which triggers polymerization inhibition, we explored alternative PIs for this efficient lithography technique. We identified 4,4′-bis(diethylamino)benzophenone (BDEBP) as a novel candidate for TSA lithography (**Figure** **4**a and b). Notably, this PI has been used in another work^15^ reporting that, when BDEBP is combined with diffusing radical quenchers, structures can be fabricated with a resolution exceeding conventional DLW systems. Both our theoretical and transient absorption studies revealed (T_1_→T_*n*_) transitions between 500 and 800 nm. For this reason, we tested a switching beam in such spectral range (642 nm) verifying the successful polymerization inhibition process by TSA (see inset of Figure 4d). This example clearly shows the general validity of the identified phenomenon and the great potential of the investigative methodology undertaken in this work.

To summarize, we have presented a comprehensive study on the photophysical mechanism responsible for polymerization inhibition, which represents the key for achieving DLW lithography with sub-diffraction resolution. By combining the study of molecular dynamics together with theoretical calculations we demonstrate step-by-step that a PA in the triplet manifold of the PI is the photophysical transition associated to the polymerization inhibition. Importantly, TSA lithography does not have to be necessarily limited to DLW lithography but can be extended to other conventional lithography techniques improving their resolution and cost effectiveness. In mask lithography, for instance, it is foreseen that implementing simultaneous exposure of the photoresist with proper diffraction-limited overlapping patterns at excitation and switching wavelengths can result in an immediate increase in resolution. Depending on the structure density, multiple exposures (up to *n* times for each direction corresponding to *n* times resolution enhancement) must be used for the fabrication of the final pattern. The possibility of increased resolution provided by the UV-vis TSA technique can indeed offer a very interesting alternative to the extremely challenging and costly extreme UV (XUV or EUV) lithography or multiple deep-UV patterning approaches to comply with Moore's Law.^35^ Our study has highlighted the advantages of the presented TSA lithography technique demonstrating that it has the potential to bring the field of DLW lithography into new dimensions. It also underlines the unquestionable necessity of having exact knowledge of the photophysical processes involved in the inhibition mechanisms. Through a detailed theoretical and spectroscopic analysis of the PIs we have shown that the phenomenon we discovered can be well rationalized by a number of crucial parameters, e.g., lifetime, transition energy, and oscillator strength of the photoinduced species. This indeed provides a correct control of each pathway involved, a reduction of any undesirable loss, and a screening procedure for exploring new candidate materials or switchable molecular systems opening the way for a broader application of this promising technology.
