# Supplementary material for: Polymerization Inhibition by Triplet State Absorption for Nanoscale Lithography
Source: Adv Mater. 2013 Jan 9;25(6):904–9. doi: 10.1002/adma.201204141 (PMC3594812; doi:10.1002/adma.201204141)
Supplement: Supplementary file 1 [file adma0025-0904-sd1.pdf]

Copyright WILEY-VCH Verlag GmbH & Co. KGaA, 69469 Weinheim, Germany, 2013.

ADVANCED MATERIALS

Supporting Information

for *Adv. Mater.*, DOI: 10.1002/adma.201204141

Polymerization Inhibition by Triplet State Absorption for Nanometer-Scale Lithography

Benjamin Harke, William Dallari, Giulia Grancini, Daniele Fazzi, Fernando Brandi, Annamaria Petrozza,* and Alberto Diaspro*

SUPPORTING INFORMATION

Sample preparation

The photoinitiator molecules investigated in our work are:

- 7-diethylamino-3-thenoylcoumarin (DETC, Exciton, USA),
- isopropyl thioxanthone (ITX, Sigma Aldrich),
- 4,4'-bis(diethylamino)benzophenone (BDEBP, Sigma Aldrich),
- phenylbis(2,4,6-trimethylbenzoyl)phosphine oxide (BAPO, Sigma Aldrich) also known as irgacure 819.

The monomer used for all polymerization experiments was pentaerythritol triacrylate (PETA, Sigma Aldrich). The different photoresins were prepared by simple diluting ITX (0.16 wt%), DETC (0.25 wt%), BDEBP (0.25 wt%), and BAPO (0.32 wt%) in PETA (2 mg). For lithography experiments, a drop of the resin was cast on a regular glass cover slip and rinsed after exposure in methanol and isopropanol for few tens of seconds. For the transient absorption measurements, few milliliters of the resin was placed in a regular glass cuvette.

Nanosecond Transient Absorption

Nanosecond transient absorption measurements were carried out with a LP920 laser flash spectrometer (Edinburgh Instruments). It is based on a standard "pump-probe" setup where the sample is excited by a nanosecond laser pulse (pump) and the time evolution of the differential absorption changes induced by the pump is monitored by a second weak probe generated by a CW light source. The pump pulses are provided by a nanosecond tunable OPOLett-355II laser (10 Hz repetition rate). The probe light is provided by a pulsed Xenon arc lamp. The sample was kept at a 45° angle to the excitation beam. The beams are focused onto the sample ensuring the spatial overlap. The transmitted probe is spectrally filtered by a monochromator and detected. Two different detection systems are used: a cooled ICCD camera which enables to detect the entire spectral range from 350 to 850 nm at once and a set of photomultipliers (with both VIS and near-IR detection window) enabling one to collect the single-wavelength kinetic with higher sensitivity. The signal is finally recorded by recorded by a TDS 3032C digital signal analyzer. From the transmission change following photoexcitation the variation in the absorption is thus derived as:

$$\Delta OD(\tau,\lambda) = \frac{\log(I_{probe})}{I_t(\tau,\lambda)} \quad \textbf{(1)}$$

where I_{probe} is the transmitted probe with excitation off and I_t is the transmitted probe after laser excitation. The system has sensitivity of $5 \cdot 10^{-4}$ and a temporal resolution of 7 ns.

Optical Setup

The optical setup used is based on a custom-made optical microscope which has been previously described.[1] For polymerization experiments, a 405-nm CW laser (Cube, Coherent, USA) generates one-photon excitation light, while a Titanium-Sapphire laser (Chameleon, Coherent, USA) delivering pulses of ~150 fs at a repetition rate of 80 MHz at the laser output is used for two-photon excitation. A CW fiber laser (MPBC communications, Canada) emitting light of 642 nm acts as the inhibition light in our experimental setup. All beams go through an appropriate combination of dichroic mirrors (AHF Analysentechnik, Germany) and reach a fast galvanometer mirror pair (Cambridge Technologies, USA) which are placed in the conjugated plane of the back-aperture of the objective lens (PL APO 100x/0.7-1.4, oil immersion, Leica Microsystems, Germany) by using a scan and tube lens

combination (Leica Microsystems, Germany). The software package Imspector (Max-Planck Innovations, Germany) ensures the full control of the optical setup.

Computational methods
Quantum chemical calculations have been carried out in the frame of density functional theory (DFT) and time-dependent (TD)-DFT. Each molecular structure has been fully optimized in both the ground singlet state (restricted DFT - RDFT), namely S_0, and the triplet (unrestricted DFT-UDFT) state, namely T_1. PBE0 DFT exchange-correlation functional[2] within a double split Pople basis set, 6-311G**, has been adopted. Singlet and triplet excited states, namely vertical excitation energies, oscillator strengths and CI expansion coefficients, have been computed with TD-PBE0 and TD-UPBE0 approaches. Ground singlet- and triplet-state force fields have been calculated to check the equilibrium stability of the optimized structures and no imaginary frequencies have been obtained for each molecule studied. All calculations have been carried out by using the Gaussian09 code.[3]

Point Pattern
A periodic point pattern was written as described in the main text. The full data set of the SEM images is shown in **Figure S1**. All fabrication parameters are the same as already described.

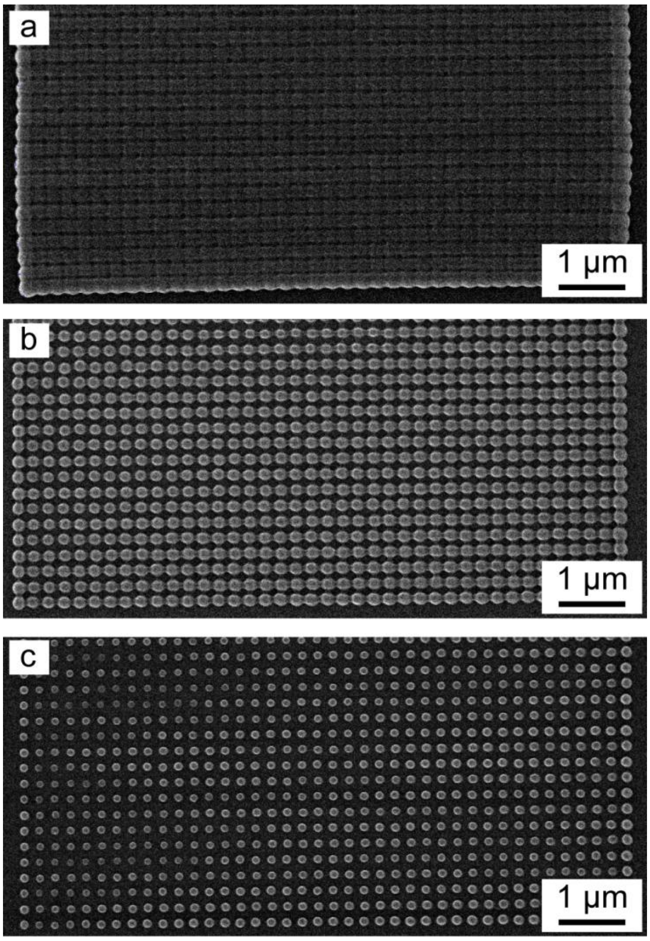

Figure S1: Periodic point pattern with a centre-to-centre distance of 250 nm fabricated by conventional DLW lithography (a) and by TSA lithography using two different switching powers: 14 mW (b) and 48 mW (c). The excitation power was kept constant for each pattern (7 mW). All power values were measured at the back aperture of the objective lens.

[1] B. Harke, P. Bianchini, F. Brandi, A. Diaspro, *ChemPhysChem* **2012**, *13*, 1429–1434.

[2] C. Adamo, V. Barone, *Chemical Physics Letters* **1999**, *314*, 152–157.

[3] M. J. Frisch, G. W. Trucks, H. B. Schlegel, G. E. Scuseria, M. A. Robb, J. R. Cheeseman, G. Scalmani, V. Barone, B. Mennucci, G. A. Petersson, others, *Wallingford CT* **2009**.
